# PLS and N-PLS based MIA-QSPR modeling of the photodegradation half-lives for polychlorinated biphenyl congeners

**DOI:** 10.1039/d0ra05231k

**Published:** 2020-09-11

**Authors:** Nasser Jalili-Jahani, Azadeh Fatehi, Ehsan Zeraatkar

**Affiliations:** a Green Land Shiraz Eksir Chemical and Agricultural Industries Company Shiraz 7137753451 Iran nj.jahani.chem@gmail.com +98 7132232636 +98 7132232636; b Shiraz Urban Railway Organization Shiraz 7193689711 Iran

## Abstract

Multivariate image analysis applied to quantitative structure–property relationships (MIA-QSPR) has been used to predict photodegradation half-lives of polychlorinated biphenyls in *n*-hexane solution under UV irradiation. Owing to the high cost and laboriousness in experimental tests, developing a simple method to assess the photostability of the compounds is important in environmental risk assessment. The predictor block was built by superposition of the chemical structures (2D images), which was unfolded to a matrix, suitable for multilinear and classical partial least squares, N-PLS and PLS, respectively, as regression methods, demonstrating different predictive capability to each other. Model performance was improved after removing an outlier, and the results were in general more accurate than the ones previously obtained through quantum chemical descriptors analysis. Model validation and *Y*-randomization test proved that the developed model has goodness-of-fit, predictive power, and robustness. Additionally, the applicability domain of the developed model was visualized by Williams plot. This study showed that a simple procedure is able to give highly predictive models, useful in ecotoxicology, independent of the regression method used for this class of compounds.

## Abstract 2

Multivariate image analysis as a useful tool in environmental risk assessment studies.

## Graphical abstract

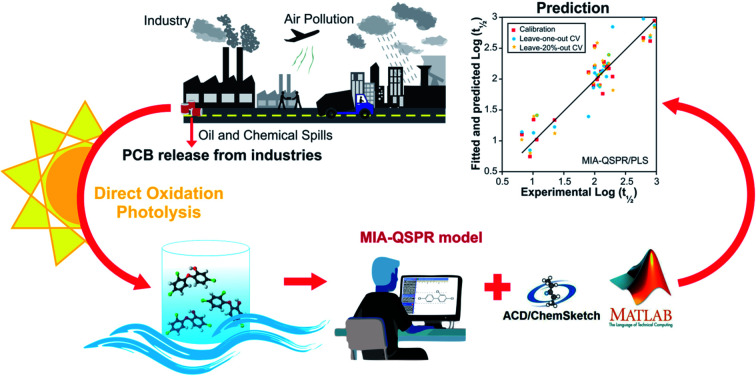

## 1 Introduction

Polychlorinated biphenyls (PCBs) are highly persistent, lipophilic and bioaccumulative toxic industrial chemicals that occur as environmental contaminants.^1,2^ Although use of these ubiquitous contaminants has been banned in industrialized countries since the late 1970s, their continued presence in the environment poses considerable hazards.^3,4^ Recent studies have revealed that many PCBs are endocrine disrupting chemicals, *i.e.* they are exogenous substances that cause adverse health effects on an intact organism or its progeny, consequent to changes in endocrine function.^4–6^ Moreover, the hydrophobicity and inertness of PCBs suggest that they can undergo long-range transport and be deposited into aquatic systems, especially sediments, where they can bioaccumulate in food chains.^7–9^

Photolysis may be one of major abiotic transformations of the chemicals in the environment. Many investigations on the photodegradation of PCBs have been reported in recent decades.^10–13^ PCBs are photosensitive to UV irradiation in aqueous and organic solutions.^10–13^ Photochemical behaviors of PCBs in organic solutions have been reported by many researchers. For example, photochemistry of PCBs was investigated in cyclohexane,^14^ in *n*-hexane,^15,16^ or in alkaline 2-propanol.^17–20^ Dechlorination appears to be the major photochemical reaction of PCBs. Reportedly, highly chlorinated PCB congeners particularly those with substitutions at the *ortho* positions are most vulnerable to photochemical attack.^21^ Previous studies indicated that photoreactivities were lower in symmetrical and coplanar PCB congeners, and reactivity was in the order of chlorine at *ortho*- > *meta*- > *para*-positions of PCB rings under UV irradiation in *n*-hexane.^15,16^ Chang *et al.* studied the photolysis of 7 PCBs in water under 254 nm UV irradiation and revealed that photodechlorination of PCBs in water is similar to that in *n*-hexane.^11^

Because photochemical transformation is often suggested as a potentially important fate process for PCBs, photodegradation half-life is one of the most important parameters and is indispensable for environmental risk assessment of these chemicals. However, measured data are rather scarce regarding photodegradation half-lives of PCBs in *n*-hexane because of large expenditures of money, time, and equipment.^10,12^ Thus, it is of great importance to develop quantitative structure–property relationship (QSPR) relating photodegradation process data to other physicochemical properties or structural descriptors. When significant QSPR models are obtained, they may provide insight into which aspect of the molecular structure influences the property.^22–25^ Moreover, they may also enable simple and fast estimation of photodegradation process and generate predicted photodegradation process data efficiently for these compounds. Niu *et al.* conducted a QSPR study on photodegradation half-lives of 22 individual PCBs in *n*-hexane solution under UV irradiation.^26^ Establishment of their model was implemented based on the calculation of quantum chemical descriptors and partial least squares (PLS) regression. Among five descriptors used in this model, standard heat of formation, total energy and molecular weight had a significant effect on photodegradation half-lives of PCBs. Model statistical tests, which show its predictive power, led to a parameters correlation coefficient of *r*^2^ = 0.659, cross-validated correlation coefficient of *q*^2^ = 0.589, and standard error of SE = 0.357.

MIA-QSPR (multivariate image analysis applied to quantitative structure–property relationship) modeling is another technique applied to predict properties, providing models with satisfactory predictive capability.^27^ Its main advantage over most of the structure based methodologies lies on the no need for conformational screening and 3D alignment; it just requires a 2D alignment, which refers to simply superimpose 2D images–2D chemical structures drawn with the aid of an appropriate drawing program.

In most of the QSPR studies, PLS is the main regression method applied to correlate descriptors with the corresponding dependent variables.^28^ However, multilinear PLS (N-PLS) is supposed to be superior to the unfolding PLS due to its simplicity (the number of variables can be effectively reduced) and predictive ability.^29,30^ This study is devoted to building a reliable MIA-QSPR model for estimating photodegradation half-life of PCBs in *n*-hexane. The goal of our work is to compare the abilities of prediction from PLS and N-PLS regressions. The comparison has been carried out such that the best performance of each method is compared.

## 2 Theoretical backgrounds

### 2.1 Partial least squares

The two-way PLS model consists of two groups of variables, commonly referred to as the predictor *X* and the response *Y*. The goal is to successively find orthogonal linear combinations of the predictor and response variables, known as predictor/response scores, that account for as much as possible of the covariation between *X* and *Y*. Specifically, PLS model can be represented by two outer relations that decompose the data blocks into sum of components:1
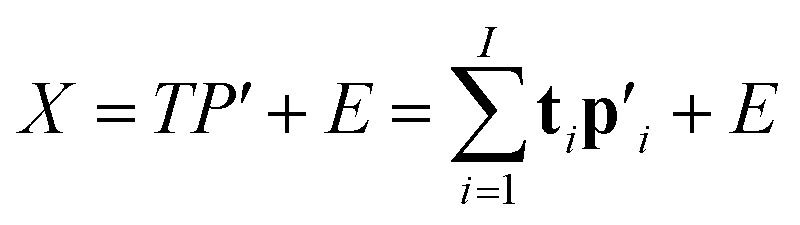
2
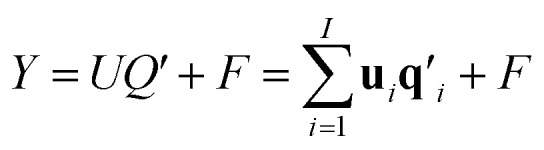
and an inner relation which ensures the maximal covariance between scores for each component

3 **u**_*i*_ = *b*_*i*_**t**_*i*_ + *e*_*i*_, *i* = 1, …, *I*

where *I* denotes the total number of PLS components. The vectors **t**_*i*_ and **u**_*i*_ are the scores of the *i*^th^ PLS component for *X* and *Y*, respectively. **p**_*i*_ and **q**_*i*_ are the associating normalized loading vectors. *b*_*i*_ is the regression coefficient for the *i*^th^ component. *E* and *F* are residual matrices. The *Y*-residuals *F* express the deviations between the observed and modeled responses. The number of components needed to describe the data blocks can be determined based on the amount of variation that remains in the residual matrices.^31^

Estimation of the PLS model is done in sequential fashion, component by component. The estimation starts with a random initialization of the response score **u**_1_. This vector is regressed on the predictor block *X* to give the block weight 
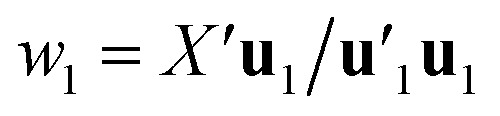
, which is normalized and multiplied with *X* to give the predictor score **t**_1_ = *X***w**_1_. For the response variables, the regression is done similarly: **t**_1_ is regressed on *Y* to yield block loading vector 
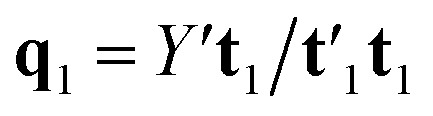
 and a new 
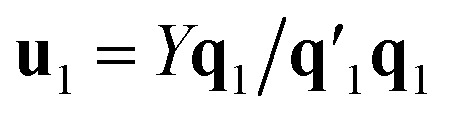
. This is repeated until **t**_1_ and **u**_1_ converge to a predefined precision, *i.e.*, ‖**t**_old_ − **t**_new_‖/‖**t**_new_‖〈*ε*, where *ε* is “small”, *e.g.*, 10^−6^ or 10^−8^. After convergence, loading vector **p**_1_ is calculated by regressing **t**_1_ on *X* and the data blocks are deflated by subtracting 
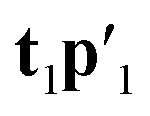
 from *X* and 
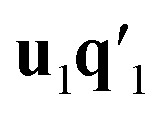
 from *Y*. The second pair of PLS components, orthogonal to the first, can be determined by setting *E* = *X* and *F* = *Y* and repeating the iteration until cross-validation indicates that there is no more significant information in *X* about *Y*. The complete algorithm for estimating two-way PLS model is given in Geladi and Kowalski.^31^

### 2.2 Multilinear partial least squares

The multilinear PLS models are called N-PLS models in general. N-PLS is an algorithm of the PLS family adapted to multimodal data (tensor variables). Tensors, or multi-way arrays, are higher order generalizations of vectors and matrices. Elements of a tensor *X̲* ∈ **R**^*I*_1_×*I*_2_×…× *I*_*N*_^ are denoted **x**_*i*1,*i*2,…,*i*N_. Here, N is the order of the tensor, *i.e.*, the number of dimensions, also known as ways or modes. Bilinear PLS can cope with multi-way data by unfolding the data arrays to matrices along the *i*^th^ mode,^32^ but the method itself is not multi-way and do not take advantage of any multi-way structure in the data.^33^ Unfolding can be unfavorable for several reasons: (1) unfold models are complex (many parameters), (2) unfold models are difficult to interpret (confounding of modes), (3) multi-way information is thrown away, and (4) risk of poor predictive power.

N-PLS models a linear relationship between input (*X̲*) and output variables (*Y*). The goal of the algorithm is to make a decomposition of the array *X̲*(*I* × *J* × *K*) into triads similar to the PARAFAC (parallel factor analysis) model. A triad consists of one score vector (**t**) and two weight vectors; one in the second mode called **w**^*J*^ and one in the third mode called **w**^*K*^.^34^ N-PLS is not fitted in a least squares sense but seeks in accordance with the philosophy of PLS to find a set of weight vectors **w**^*J*^ and **w**^*K*^ that produces a score vector (**t**) with maximal covariance with *Y*.^34^ This is obtained by making a matrix **Z** = *X̲′***u**_1_, by decomposing the matrix *Y* into one score vector **u**_1_ and one weight vector **q**_1_, and then decomposing **Z** by SVD (singular value decomposition) into two loading vectors **w**_1_^*J*^ and **w**_1_^*K*^, which, normalized, then determines the score vector **t**_1_ = *X***w**_1_ as the least squares solution.^34^ Where *X* is the array *X̲* unfolded to an (*I* × *JK*) matrix and **w**_1_ = **w**_1_^*J*^ ⊗ **w**_1_^*K*^. The symbol ⊗ denotes the Kronecker product.^35^ The coefficient **b**_1_ of regression is calculated as 
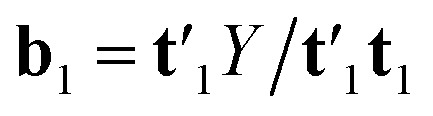
. After convergence, the data blocks are deflated by subtracting **t**_1_**w**_1_^1^(**w**_1_^2^)′ from *X̲* and **t**_1_**b**_1_ from *Y*. Then, factors are calculated in the same way by setting *E̲* = *X̲* and *F* = *Y* and applying the procedure iteratively to the residuals. A detailed description of N-PLS can be found, for example, in literature.^29,36^

### 2.3 Applicability domain

A common way to show the scope and limitations of a QSPR model, *i.e.* the range of structural information (parameters) and activities/properties of the structures is checking the applicability domain (AD) by the aid of leverage. The leverage provides a measure of the distance of the each sample from the centroid of the model space and as another word, indicates multivariate normality of observations. Compounds close to the centroid are less influential in modeling process.

The leverages or hat value (*h*_*i*_) of the *i*^th^ compound in the descriptor space was computed as below:^37^4
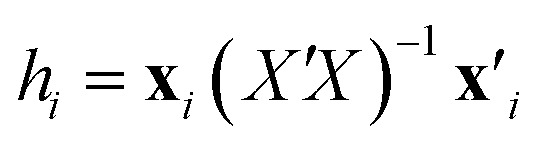
where *X* is the descriptor matrix of the training set and **x**_*i*_ is the descriptor row vector of the desired compound (in training or test set). If a mixture obtains a leverage lower than the warning value, this mixture is in the AD. The warning leverage (h* or 3*h*) is defined as h* = 3*p*/*n*, where *n* is the number of training mixtures, and *p* is the number of model variables plus one.

It should be noted that the leverage is not an enough factor to judge about the AD. In addition to the high leverage values, compounds may also fall outside the AD, because of their large “standardized residuals”.^38^ A Williams plot considers both leverage and standard residual.^38^

## 3 Experimental

### 3.1 Data set

A data set consisted of photodegradation half-life for 22 PCB congeners with 2–5 chlorinated substitutions (including 21 non-coplanar *ortho* substituted and one non-*ortho* substituted) in *n*-hexane solution was obtained from the literature^10^ to assess the performance of the N-PLS and PLS models. According this reference, each test solution (100 mL) containing an individual PCB congener (2 μg mL^−1^ in *n*-hexane) was irradiated under a 15 W UV lamp at a wavelength of 254 nm in a separate glass beaker. The chemical structures of these compounds and their photodegradation half-lives have been listed in Table 1 which shows a great different half-lives range from 6.6–926.4 min for different PCBs in this study. Thus, the original data were converted to the logarithmic scale (log(*t*_1/2_)) before analysis. Moreover, it appears that the PCB congeners with two chlorines substituted are photodegraded in 6.6–22.2 min, but if more than three chlorines are present, the photodechlorination of PCB needed 79.2–926.4 min, indicating the half-lives are affected by the molecular size.

**Table 1:** Half-life of PCB congeners upon exposure to UV radiation (254 nm) in *n*-hexane solution

PCB no.	Structure	Half-life (min)
4	2, 2′	10.2 ± 2.2
5	2, 3	9.0 ± 2.2
6^a^	2, 3′	11.4 ± 1.6
8	2, 4′	6.6 ± 2.1
10	2, 6	22.2 ± 3.3
17	2, 2′, 4	96.6 ± 18.3
18	2, 2′, 5	164.2 ± 11.1
19	2, 2′, 6	196.2 ± 15.7
27^a^	2, 3′, 6	135.0 ± 20.9
34	2′, 3, 5	100.2 ± 7.8
47	2, 2′, 4, 4′	926.4 ± 11.8
49	2, 2′, 4, 5′	100.2 ± 12.5
50^a^	2, 2′, 4, 6	170.4 ± 23.2
51	2, 2′, 4, 6′	139.8 ± 12.1
52	2, 2′, 5, 5′	802.8 ± 47.7
53	2, 2′, 5, 6′	124.8 ± 5.3
62	2, 3, 4, 6	121.2 ± 35.2
73	2, 3′, 5′, 6	154.8 ± 20.9
104	2, 2′, 4, 6, 6′	115.2 ± 17.4
118	2, 3′, 4, 4′, 5	79.2 ± 5.2
121^a^	2, 3′, 4, 5′, 6	110.4 ± 6.2
126	3, 3′, 4, 4′, 5	621.0 ± 7.5

a Test set.

### 3.2 Model development

**Fig. 1:**
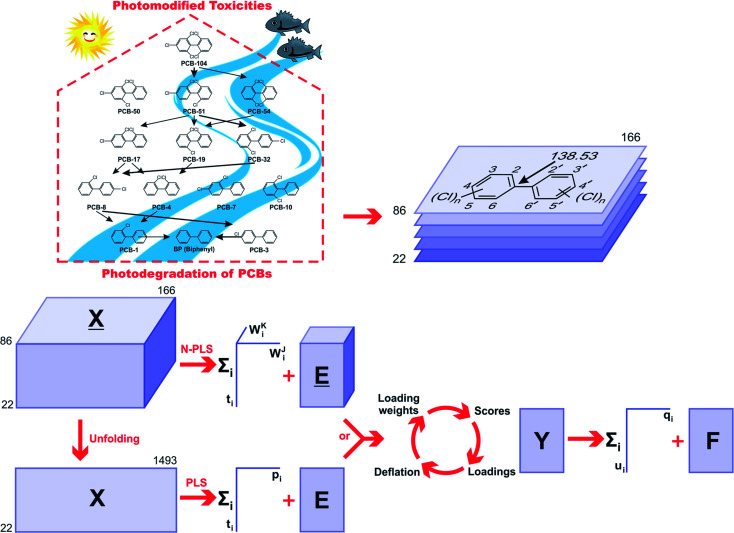
Dechlorination pathways of PCBs in water under UV irradiation [10,11]. Image superposition, building of the three-way array (*X̲*, suitable to N-PLS regression), and unfolding to a two-way array (*X*-matrix, suitable to PLS regression). The arrow in the molecular structure indicates a pixel in common among the whole set of images (2D chemical structures) fitted at the 138.53 coordinated used for the 2D alignment step.

The 2D chemical structures were built using the ChemSketch program,^39^ then aligned by a common pixel among them in a defined workspace of dimension 150 × 200 pixels (Fig. 1), and finally saved as bitmaps. According to Fig. 1, the 22 images were read as double arrays in Matlab^40^ and aligned to give a 22 × 150 × 200 three-way array (*X̲*). The lateral and frontal slices indicating no variances were removed from the three-way data. This process gave a three-way array of 22 × 86 × 166 dimension, which was regressed against the *Y*-block through N-PLS. Then, the three-way data was unfolded to a two-way data *X*-matrix of 22 × 14 276 dimension. The size of the matrix was reduced (22 × 1493) after removing columns indicating no variances as a blank workspace or congruent structures. Subsequently, the *X*-matrix was regressed against the *Y*-block through PLS. The superposition of congruent structural scaffolds, the generation of the three-way array and the unfolding step are illustrated in Fig. 1. The statistical parameters used to evaluate the model performances were the root mean square errors of calibration (RMSEC), leave-one-out (LOO) cross-validation (RMSECV),^28^ and leave-20%-out (L20%O) cross-validation (RMSECV_20%_),^28^ and the squared correlation coefficients of the regression lines of experimental *vs.* fitted (*r*^2^) and predicted (*q*^2^ or *q*_20%_2).^28^

## 4 Results and discussions

The photodegradation half-life of a pesticide can give scientists an indication of how easily a pesticide might be photodegradated under sunlight irradiation in natural surface waters, and is useful for assessment of its toxicity to animals and aquatic life. This parameter is usually represented by the logarithmic scale (log(*t*_1/2_)), which may be easily estimated through calculations. However, we have found that a simple correlation result between calculated molecular structural descriptors (such as constitutional descriptors, electrostatic descriptors, topological descriptors, geometrical descriptors and quantum chemical descriptors), and log(*t*_1/2_) for the 22 title-based compounds was very poor.^26^ Therefore, MIA-QSPR arises as an alternative method to derive useful models without having to proceed with conformational screening and 3D optimization. The log(*t*_1/2_) values for the 22 PCBs used in development of the PLS and N-PLS models are listed in Table 1.

**Fig. 2:**
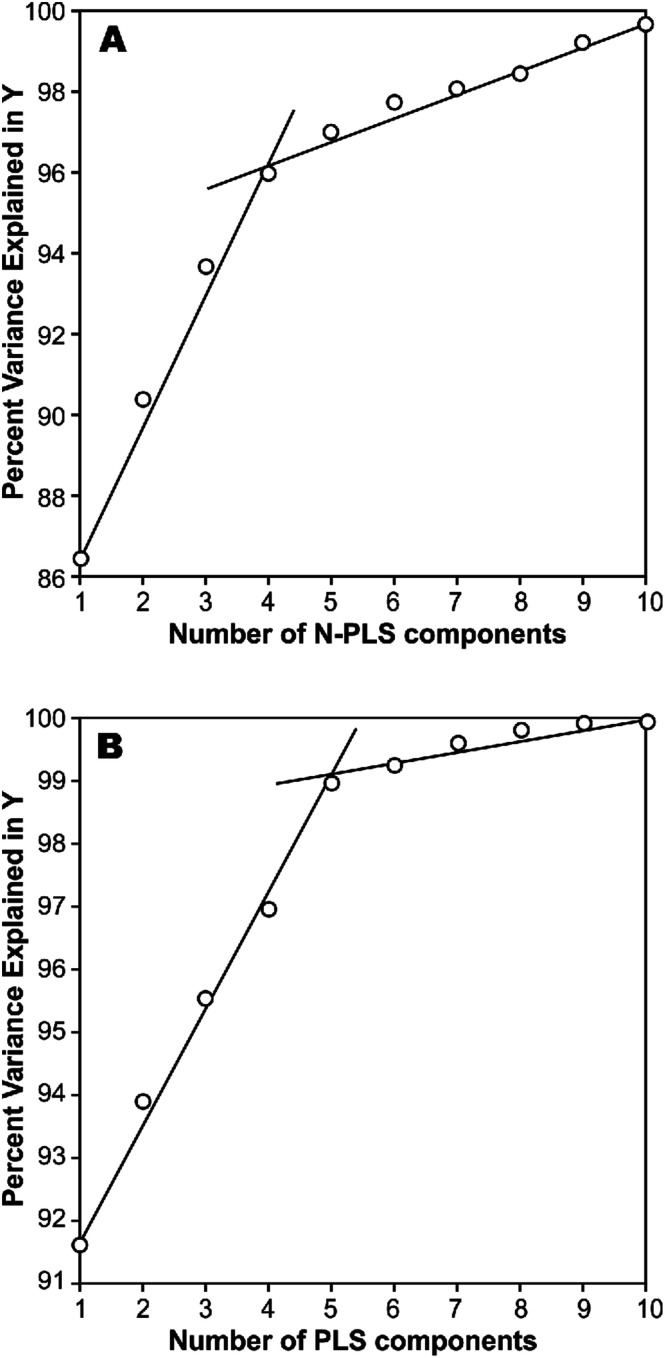
Influence of number of (A) N-PLS and (B) PLS components added on total variance explained in *Y*-block.

In any empirical modeling, it is essential to determine the correct complexity of the model. With numerous and correlated *X*-variables there is a substantial risk for a “over-fitting”, *i.e.*, getting a well fitting model with little or no predictive power. Hence, a strict test of the predictive significance of each N-PLS or PLS component is necessary, and then stopping when components start to be non-significant. In this study, the best number of latent variables was searched using the break-point algorithm to avoid over-correlation of the regression equations.^41^ This procedure shows the break-point (the change in the slope) in the plot of percent variance explained in *Y versus* the number of components added (Fig. 2).

**Fig. 3:**
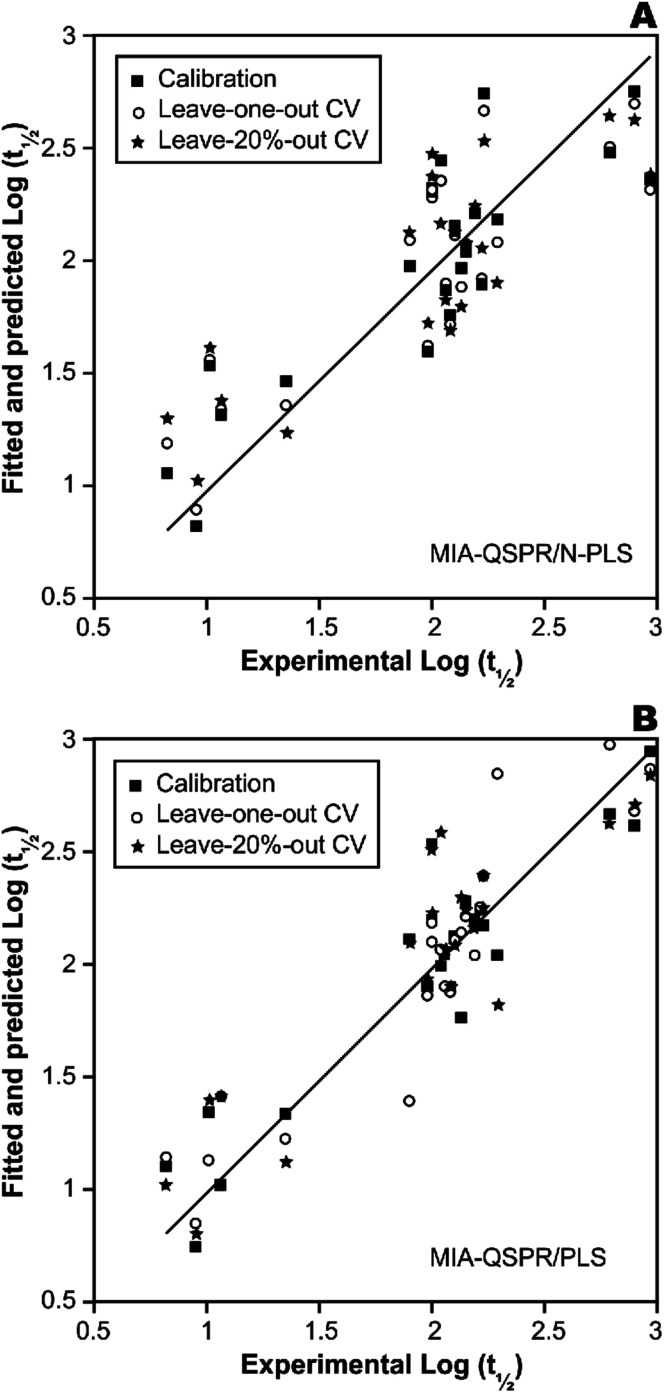
The scatter plots of experimental *vs.* calibrated and predicted the logarithmic scale half-life values for the (A) N-PLS and (B) PLS based MIA-QSPR models built.

In a first approach, an MIA-QSPR model was built using N-PLS to correlate the three-way array *X̲* (the descriptors block) with the log(*t*_1/2_) values. Four N-PLS components were found to be optimum using the break-point algorithm (Fig. 2(A)). A reasonable *r*^2^ of 0.732 (RMSEC = 0.300) was achieved, where *r*^2^ of 0.659 was obtained using quantum chemical descriptors in the previous study.^26^ The N-PLS based model was validated through LOO cross-validation, in which 22 models were developed with one different prediction sample at a time; a *q*^2^ of 0.718 (RMSECV = 0.310) was obtained. LOO cross-validation has often been considered to be an incomplete validation method; external validation has been strongly recommended instead.^42^ Randomly selected samples, 20% from the total series of 22 compounds, were also used as the external test set. Randomization was performed 10 times, and an average *q*_20%_2 was considered, *i.e.* 0.699 (RMSECV_20%_ = 0.321). The estimation and prediction using MIA-QSPR/N-PLS are shown in Table 2 and illustrated in Fig. 3(A).

**Table 2:** Experimental, calibrated, cross-validated, and predicted photodegradation half-lives (log(*t*_1/2_)) of polychlorinated biphenyls

PCB no.	Exp.	MIA-QSPR/N-PLS			MIA-QSPR/PLS			MIA-QSPR/PLS	
		Cal.	LOO	L20%O	Cal.	LOO	L20%O	Cal.	Predicted
4	1.01	1.53	1.58	1.62	1.35	1.16	1.40	1.36	
5	0.95	0.82	0.89	1.03	0.75	0.87	0.81	0.85	
6	1.06	1.31	1.35	1.37	1.03	1.43	1.41		1.15
8	0.82	1.06	1.20	1.30	1.11	1.17	1.02	1.01	
10	1.35	1.46	1.37	1.24	1.34	1.25	1.12	1.50	
17	1.98	1.60	1.64	1.72	1.90	1.88	1.93	1.68	
18	2.22	1.90	1.92	2.05	2.24	2.26	2.24	1.84	
19	2.29	2.18	2.08	1.90	2.04	2.86	1.82	2.20	
27	2.13	1.97	1.88	1.80	1.76	2.16	2.29		1.94
34	2.00	2.31	2.32	2.47	2.53	2.19	2.50	2.32	
47	2.97	2.36	2.31	2.37	2.93	2.88	2.83	2.55	
49	2.00	2.31	2.30	2.37	2.18	2.12	2.22	2.12	
50	2.23	2.74	2.66	2.53	2.17	2.40	2.39		2.70
51	2.15	2.04	2.07	2.07	2.28	2.23	2.24	2.08	
52	2.90	2.75	2.70	2.62	2.61	2.69	2.70	2.68	
53	2.10	2.15	2.11	2.15	2.12	2.12	2.08	2.25	
62	2.08	1.76	1.72	1.70	1.90	1.90	1.90	1.97	
73	2.19	2.21	2.22	2.24	2.20	2.06	2.16	1.73	
104	2.06	1.87	1.90	1.83	2.04	1.92	2.06	2.01	
118	1.90	1.97	2.10	2.12	2.11	1.41	2.09	2.14	
121	2.04	2.44	2.35	2.16	1.99	2.08	2.58		2.30
126	2.79	2.48	2.51	2.64	2.66	2.99	2.61	2.75	

The three-way array used for the N-PLS treatment was unfolded to a two-way array, an *X*-matrix of dimension 22 × 1493 suitable to be regressed against the log(*t*_1/2_) values through classical (bilinear) PLS. Fig. 2(B) reveals the notion that increasing the number of parameters only up to five has a large influence on total percent variance explained in *Y*. The calibration using five PLS components gave an *r*^2^ of 0.871 (RMSEC = 0.208) (Table 2 and Fig. 3(B)), which is superior to the correlation found in the literature.^26^ The calibration model was validated by LOO and L20%O cross-validations, giving *q*^2^ of 0.857 (RMSECV = 0.226) and *q*_20%_^2^ of 0.819 (RMSECV_20%_ = 0.254). A comparison of the developed models shows that the MIA-QSPR/PLS model can simulate the relationship between obtained descriptors by MIA and the log(*t*_1/2_) values of studied PCBs more accurately. Unfortunately, the compounds in model only contain PCB congeners with 2–5 chlorinated substitutions due to a lack of experimental data. The model is thus limited due to its domain of application.

Next, the whole data set was in fact randomly split into training (80% of the whole set of compounds) and test sets (one randomly selected among the DiCBs, TriCBs, TetraCBs, and PentaCBs, respectively), as depicted in Table 1, in order to give insight about a real external validation; five PLS components were found to be better, and *r*^2^ of 0.842 and *r*_test_^2^ of 0.829 were achieved. According to Table 2, increasing molecular graph of the PCBs leads to increase of the log(*t*_1/2_) values. As all the PCB molecules have a same parent biphenyl, it can be concluded that the more chlorine atoms in the parent molecule, the higher the log(*t*_1/2_) values. This conclusion is consistent with the result from Chang *et al.*, who found that photodegradation rates of PCB congeners decreased with the increasing of chlorides in the biphenyl.^10^ The results also are similar to the observations of Chen *et al.*, who reported QSPR models on direct photolysis of them dissolved in water : acetonitrile solution.^22,23^ In addition, Niu *et al.* investigated photolysis of PCBs on fly ash surfaces and irradiated by UV simulated sunlight, and found the similar conclusion.^24^

Additional statistic has been proposed in order to test the external predictability, namely *r*_m_^2^ which is defined as:^43^

5 *r*_m_^2^ = *r*^2^[1 − (*r*^2^ − *r*_0_^2^)^1/2^]

where *r*^2^ and *r*_0_^2^ are the squared correlation coefficient values between observed and predicted values of the test-set compounds with and without the intercept, respectively. For a model with good external predictability, the *r*_m_^2^ value should be greater than 0.5. The *r*_m_^2^ value for the PLS model for test set was 0.803. Therefore, the model is equally predictive according to this validation method.

**Fig. 4:**
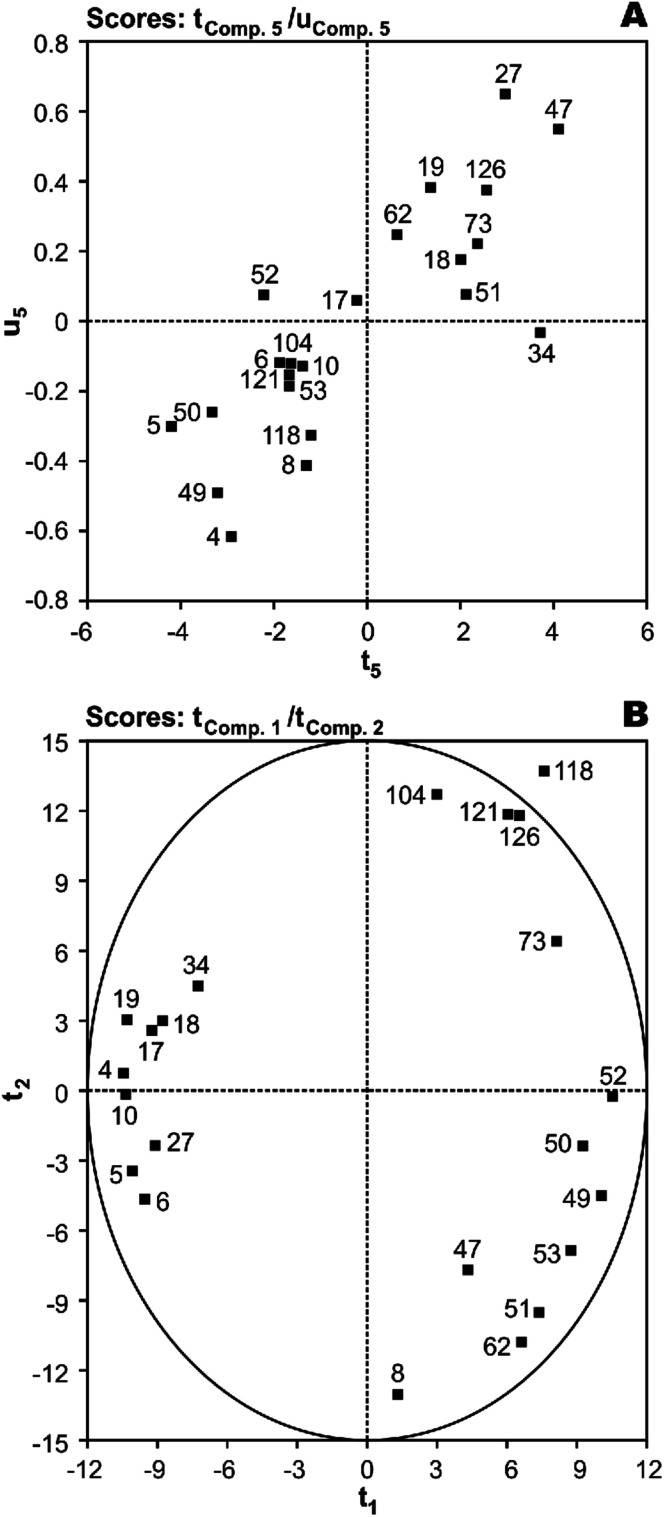
The plot of (A) **t**_5_ (*X*-score) *vs.***u**_5_ (*Y*-score) and (B) *X*-scores (**t**_1_*vs.***t**_2_) of the studied PCBs in the developed five component MIA-QSPR/PLS model.

*T*(*X*-scores) *vs. U*(*Y*-scores) plots were used for homogeneity analysis and evaluating the prediction performance of the image regression model.^44^ Homogeneity means that the investigated system or process must be in a similar state throughout all the investigation and the mechanism of influence of *X* on *Y* must be the same. With five significant PLS components (**t**_1_ ∼ **t**_5_), first, the most important factor was identified using serially correlating of each component to *Y* and the resulting values were 27.0%, 1.3%, 6.0%, 19.4%, and 32.4%, respectively. As shown, the results have rank 1 in the fifth component. The plot of *X*-score **t**_5_*vs.* corresponding *Y*-score **u**_5_ shows that only the PCB-34 may be show a much worse fit than the others, indicating an inhomogeneity in the data (Fig. 4(A)). To investigate this, a second round of analysis was made with a reduced data set, *N* = 21, without the PCB-34. The modeling of *N* = 21 PCBs with the same linear model as before gives a slightly better result with *r*^2^ of 0.892 and *q*^2^ of 0.871. Thus, this molecule cannot be assumed to be an outlier.

Score plots *T*(*X*-scores) are important to explore the distribution of molecules in the latent variable space and shows object similarities and dissimilarities.^44^ The scores obtained from first two components **t**_1_*vs.***t**_2_ are only plotted here to see the distribution of molecules and also check any outliers are present in the dataset or not. If any compound is positioned outside the ellipse (at 99% significance level), then we can consider that compound as an outlier. In the score plot, the ellipse represents the applicability domain of the PLS model developed by using PCBs as defined by Hotelling's *T*^2^. Hotelling's *T*^2^ is a multivariate generalization of Student's *t*-test.^45^ We can identify the outliers from this plot. Fig. 4(B) shows that compounds which are situated in the left hand corner bearing similar properties whereas the compounds which are far apart from each other like those situated in the lower right hand corner represent dissimilar compounds. As shown, there is not a clear overlapping point between compounds. The data separation is very important in the development of reliable and robust QSPR models. It has also been found from the Fig. 4(B) that PCB-118 is situated outside the ellipse and indicated as an outlier. This time, the above-mentioned outlier detection strategy gives a substantially better result with *r*^2^ of 0.938 and *q*^2^ of 0.925, confirming the legitimacy of PCB-118 as an outlier.

**Fig. 5:**
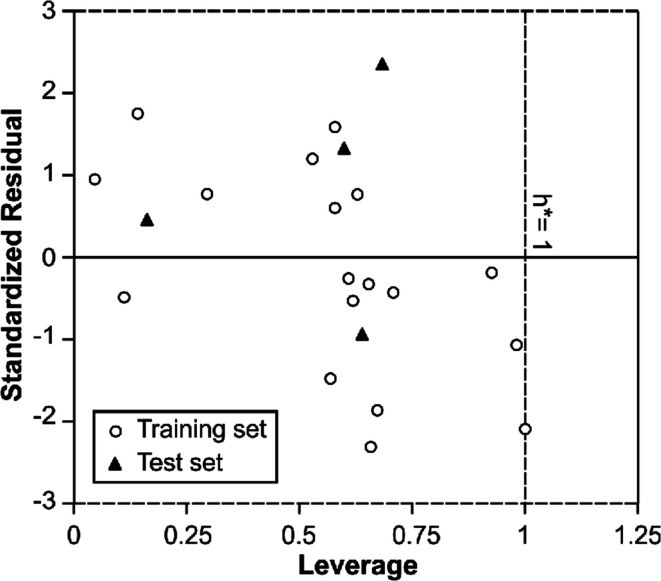
William's plot of generated PLS based MIA-QSPR model.

In order to use MIA-QSPR model to assess new chemicals, its applicability domain needs to be defined and only those predictions that fall within this domain may be regarded as reliable.^37^ The applicability domain of the developed MIA-QSPR/PLS model was validated by an analysis of the Williams graph of Fig. 5, in which the standardized residuals and the leverage value (*h*) are plotted. It can be clearly seen that all of the 22 compounds were located within the boundaries of applicability domain, which indicated that our proposed MIA-QSPR/PLS model had a well-defined AD. In addition, the random distribution of residuals on both sides of zero line indicates that there is no systematic error in the development of the MIA-QSPR/PLS model.

Moreover, the robustness of the MIA-QSPR/PLS models was further evaluated using the *Y*-randomization test in this contribution.^28^ The dependent variable vector (the log(*t*_1/2_) values) was randomly shuffled and new MIA-QSPR models were developed using the original variable matrix. The new MIA-QSPR/PLS models are expected to show a low value for *r*^2^ and *q*^2^. Several random shuffles of the *Y*-vector were performed for which the results are shown in Table 3.

**Table 3:** Obtained squared correlation coefficients of the regression lines of experimental *vs.* fitted (*r*^2^) and predicted (*q*^2^) by *Y*-randomization

Iteration	*r* ^2^	*q* ^2^
1	0.110	0.153
2	0.202	0.255
3	0.131	0.238
4	0.250	0.111
5	0.018	0.048
6	0.145	0.194
7	0.078	0.055
8	0.106	0.158
9	0.029	0.133
10	0.109	0.202

Overall, we found that N-PLS and PLS behaved very satisfactorily when applied to solve MIA-QSPR analysis for a series of 22 PCBs. Also, this work was an attempt to show that a general statement that N-PLS is better than PLS in all QSARs is inadequate, and the results are in good agreement with the ones reported in the literature.^46,47^ In fact, the MIA-QSPR/N-PLS model was slightly more parsimonious than the PLS based model (four N-PLS components used in the modeling using the whole data set against five PLS components), but the predictive ability of both models were comparable to the available data from the literature,^26^ only requiring a modest computational investment and neither conformational screening nor 3D optimization rules to achieve reliable models to predict photostability of compounds harmful to the environment.

## 5 Conclusions

In the present study, pixels of chemical structures (2D images) stand for descriptors, and structural changes account for the variance in photodegradation half-lives of PCB congeners in *n*-hexane under UV irradiation. PLS and N-PLS were applied as regression methods demonstrating greater advantage of photodegradation half-lives prediction based on PLS. The LOO cross-validated value of *q*^2^ for the optimal MIA-QSPR/PLS model is 0.857, indicating a good predictive capability for the log(*t*_1/2_) values of PCBs. The results obtained are consistent with the result from previous researchers who found that photodegradation rates of PCB congeners decreased with the increase of chlorides in the biphenyl.

## Conflicts of interest

There are no conflicts to declare.

## Supplementary Material
